# The role of miR-200 family in the regulation of hallmarks of cancer

**DOI:** 10.3389/fonc.2022.965231

**Published:** 2022-09-08

**Authors:** Klaudia Klicka, Tomasz M. Grzywa, Aleksandra Mielniczuk, Alicja Klinke, Paweł K. Włodarski

**Affiliations:** ^1^ Department of Methodology, Medical University of Warsaw, Warsaw, Poland; ^2^ Doctoral School, Medical University of Warsaw, Warsaw, Poland; ^3^ Department of Immunology, Medical University of Warsaw, Warsaw, Poland; ^4^ Laboratory of Experimental Medicine, Medical University of Warsaw, Warsaw, Poland

**Keywords:** miR-200, miR-200 family, miRNA, hallmarks of cancer, tumor progression, invasiveness, metastasis

## Abstract

MiRNAs are short non-coding RNAs that regulate gene expression post-transcriptionally contributing to the development of different diseases including cancer. The miR-200 family consists of five members, miR-200a, miR-200b, miR-200c, miR-141, and miR-429. Their expression is dysregulated in cancer tissue and their level is altered in the body fluids of cancer patients. Moreover, the levels of miR-200 family members correlate with clinical parameters such as cancer patients’ survival which makes them potentially useful as diagnostic and prognostic biomarkers. MiRNAs can act as either oncomiRs or tumor suppressor miRNAs depending on the target genes and their role in the regulation of key oncogenic signaling pathways. In most types of cancer, the miR-200 family acts as tumor suppressor miRNA and regulates all features of cancer. In this review, we summarized the expression pattern of the miR-200 family in different types of cancer and their potential utility as biomarkers. Moreover, we comprehensively described the role of miR-200 family members in the regulation of all hallmarks of cancer proposed by Hanahan and Weinberg with the focus on the epithelial-mesenchymal transition, invasiveness, and metastasis of tumor cells.

## Introduction

MiRNAs (microRNAs, miRs) are small non-coding RNAs that regulate gene expression at the post-transcriptional level. They were discovered in 1993, when Lee et al. described an antisense RNA-RNA interaction between *lin-4* transcripts complementary to the 3’ untranslated region (UTR) of lin-14 mRNA in *Caenorhabditis elegans* (1). Over the past 30 years, miRNAs have become the subject of intense research, including in the field of cancer research (2, 3). So far over 2000 human miRNAs have been described (4, 5). Numerous studies have focused on the role of miRNAs in cancer development *in vitro* and *in vivo*, as well as their potential use as diagnostic and prognostic biomarkers and therapeutic agents (6, 7).

MiRNA biogenesis is a multistep process resulting in the formation of mature single-stranded miRNA (8). It begins in the nucleus where pri-miRNA is transcribed with RNA polymerase II. MiRNAs may be monocistronic (encoded individually) or polycistronic (encoded in clusters). In the next step, pre-miRNA is generated with the participation of the Drosha complex (microprocessor) and transported to the cytoplasm where it is further processed by the Dicer complex to miRNA duplex. Only one strand of the miRNA duplex (mature miRNA) forms the miRNA-induced silencing complex (RISC) (9–11).

MiRNAs bind with their seed region to complementary sequences in the 3’ UTR of mRNAs (12). The seed region is a sequence of 2-7 nucleotides at the 5’ end of the miRNA that is responsible for mRNA binding (13). That leads to the degradation of mRNA or inhibition of protein translation (14). One miRNA may target several mRNAs, as well as an individual mRNA may be regulated by numerous miRNAs. The are several bioinformatical tools (e.g. TargetScan, Starbase) that enable the prediction of the targets of miRNAs in silico, but the result must be validated experimentally (15). Thus, miRNAs form a complex miRNA-mRNA regulatory network that influences key pathways in cells and controls all biological processes including carcinogenesis (2, 12).

**Figure 1:**
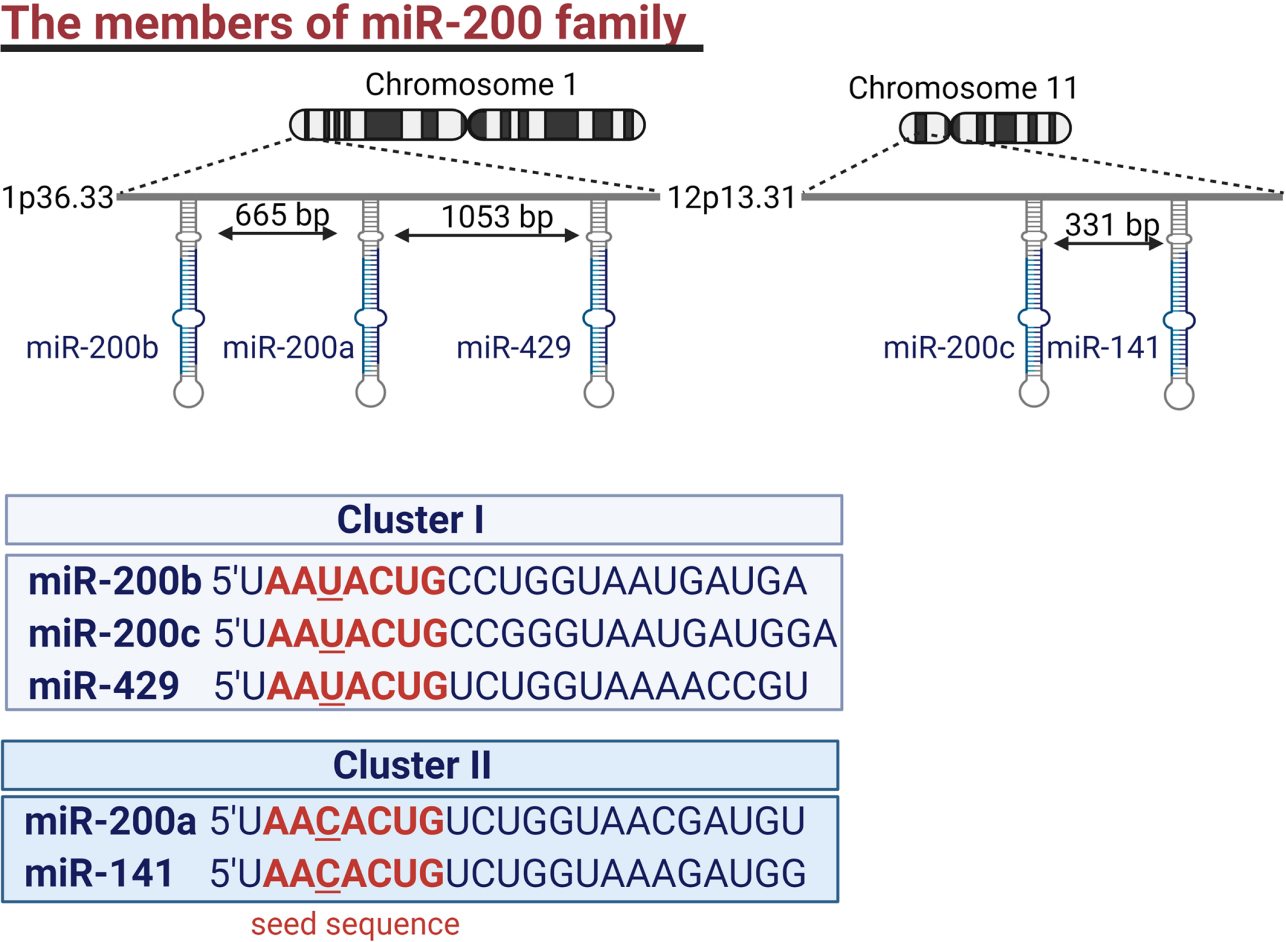
The members of the miR-200 family. Genes encoding the miR-200 family form two clusters. One encoding miR-200b, miR-200a, and miR-429 is located in chromosome 1 while miR-200c and miR-141 are encoded by genes located in chromosome 11. Members of the miR-200 family are characterized by AA(U/C)ACUG seed sequence.

The miR-200 family consists of five members, miR-200a, miR-200b, miR-200c, miR-141, and miR-429. They are divided into two clusters based on their seed sequence. MiR-200b, miR-200a, and miR-429 (cluster I) are located on chromosome 1 and have a common seed sequence (AAUACUG). While miR-200c and miR-141 belong to cluster II and are located on chromosome 11. Their seed sequence (AACACUG) differs only by one nucleotide from the seed sequence of cluster I (**Figure 1**) (16). Hence the members of the miR-200 family can theoretically target similar mRNAs (13). This review aims to comprehensively describe the role of the miR-200 family members in regulating all hallmarks of cancer.

## Expression of miR-200 family in cancer

### Dysregulation of the expression of miRNAs in cancer

MiRNA expression is often dysregulated in cancer cells. New technologies such as high-throughput RNA sequencing enable to profile miRNAs expression (17). Several studies showed the general repression of miRNAs in cancer tissues since the majority of them are tumor suppressors (18–20). One of the miRNA families that suppress cancer development and progression is the miR-200 family which is known for targeting zinc-finger E-box binding homeobox (ZEB) and inhibiting epithelial-mesenchymal transition (EMT) (21). In contrast, miR-21 is a well-known oncomiR that targets mainly tumor suppressors (22). Notably, the individual miRNA expression and functions in cancer cells may be tissue-specific and differ between different tumor types (23).

### Mechanisms of miRNAs dysregulation in cancer

The mechanisms of miRNAs expression regulation are complex and multistep (24). They may be divided into transcriptional mechanisms such as the influence of transcription factors, DNA methylation, and histone modifications (25). All of them control pri-miRNA transcription. Among numerous transcription factors that regulate miRNAs expression are p53, ZEB, Myc, and hormone receptors, including estrogen receptors (26). The next mechanism regulating miRNAs expression is DNA promoter methylation. Hypermethylation was associated with decreased expression of miRNAs, while hypomethylation caused the upregulation of miRNAs expression (27). Furthermore, there are posttranscriptional mechanisms that regulate miRNA expression by the regulation of pri-miRNA and pre-miRNA processing (26). The genes involved in miRNAs biogenesis, including DICER, are frequently dysregulated in cancer (28, 29). Downregulation of DICER is a prediction of poor prognosis for overall and progression-free survival (30).

### Expression of miR-200 family

In our review, we collected articles that determined the expression of miR-200 family members in 25 cancer types in the tumor tissue compared to corresponding healthy tissue using the RT-qPCR (real-time quantitative PCR) method (**Table 1**). MiR-200 family members were downregulated in 10 types of cancer and upregulated in 2 cancer types. In 12 types of cancer individual members of the miR-200 family had different levels of expression (**Table 1**). MiRNAs from both miR-200b/miR-200a/miR-429 and miR200c/miR-141 clusters are expressed together as two different polycistronic pri-miRNA transcripts (133). Therefore, they have a similar pattern of expression (up- or downregulation) in most cancer types. The expression of the miR-200 family is regulated by different mechanisms, including DNA methylation. miR-200b, miR-200c, and miR-141 were described as epigenetically silenced by DNA methylation in breast cancer (134). Moreover, the miR-200b/miR-200a/miR-429 cluster was repressed in the mechanism of histone modifications in this model (135).

**Table 1:** The expression of miR-200 family members in different types of cancer compared to the healthy tissue.

Cancer	miR-200a expression	miR-200b expression	miR-200c expression	miR-141 expression	miR-429 expression	Ref.
Acute myeloid leukemia	↓	↓	n/d	n/d	↓	(31)
Acute lymphoblastic leukemia	n/d	n/d	n/d	↓	↑	(32, 33)
Bladder cancer	↓/↑	↑	↓/↑	↑	↑	(34–41)
Breast cancer	↓/↑	↓/↑	↓/↑	↓/↑	n/d	(42, 43)
Cervical carcinoma	↓	↓	↓/↑	↑	↓	(44–50)
Cholangiocarcinoma	↓	↓	↓	n/d	n/d	(51, 52)
Colorectal cancer	↑	↑	↑	↑	↑/↓	(53–59)
Endometrial cancer	↑	↑	↑	↑	↑	(60, 61)
Esophageal carcinoma	↓/↑	↓	↓	↓	↓	(62–70)
Gastric cancer	↓/↑	↓/↑	↓/↑	↓	↓/↑	(71–76)
Glioma	↓	↓	↓	↓	↑/↓	(77–83)
Head and neck carcinoma	↓	n/d	n/d	↓	n/d	(84–86)
Liver cancer	↓	↓	↓	↓	↓	(87–91)
Melanoma	↓	↓	↓	↓	↓	(92–94)
Nephroblastoma	↓	↓	↓	↓	↓	(95–98)
Neuroblastoma	↓	n/d	n/d	n/d	n/d	(99)
Non-small cell lung cancer	↓	↓	↓	↑/↓	↑	(100)
Oral squamous cell carcinoma	↓	↓	↓	↓	↓	(101, 102)
Osteosarcoma	↓	↓	↓	↓	↓	(103–107)
Ovarian cancer	↑	↑	↑	↑	n/d	(108–113)
Pancreatic cancer	↑	↑	↓	↑/↓	↓	(114–118)
Prostate cancer	↓/↑	↑	↓	↑	n/d	(119–122)
Renal cell carcinoma	↓	↓	↓	↓	↓	(123–128)
Small cell lung carcinoma	n/d	n/d	n/d	n/d	n/d	n/d
Thyroid cancer	↓/↑	↓/↑	↓/↑	↓/↑	↓/↑	(129–132)

↓ - downregulated expression; ↑ - upregulated expression; n/d – no data.

### miRNAs in body fluids

Circulating miRNAs can be detected in various body fluids including serum, saliva, and urine. Thus, they are promising diagnostic and prognostic biomarkers. MiR-200 family members have been detected in different body fluids and were dysregulated in 18 cancer types (**Table 2**). The level of miR-200 family members in serum was increased in 7 cancer types and decreased in serum in 3 cancer types. There are inconsistent data concerning serum miRNAs expression in bladder cancer, prostate cancer, and non-small lung carcinoma. Notably, the expression patterns of miR-200 family members in cancer tissue are consistent with their level in body fluids only in 5 cancer types. The observation is consistent with the studies that demonstrate that the miRNAs expression profiles of patients’ serum and cancer tissue are different (194, 195). Importantly, the levels of miR-200 family in different types of body fluids vary in cancer patients and may not correlate (196).

**Table 2:** The level of miR-200 family members in the serum, urine or saliva of patients with different types of cancer.

Cancer	miR-200a level	miR-200b level	miR-200c level	miR-141 level	miR-429 level	Ref.
Acute myeloid leukemia	n/d	n/d	n/d	n/d	n/d	n/d
Acute lymphoblastic leukemia	n/d	n/d	n/d	n/d	n/d	n/d
Bladder cancer	↓ (urine)	↓ (urine)	↓ (urine)	↑/↓ (urine)	↓ (urine)	(136–139)
Breast cancer	n/d	n/d	↓	n/d	n/d	(140, 141)
Cervical carcinoma	↑	n/d	↑	↓	n/d	(48, 142, 143)
Cholangiocarcinoma	↑	↑	↑	↑	n/d	(144)
Colorectal cancer	n/d	↑	↑	↑	n/d	(145–150)
Endometrial cancer	↑	n/d	n/d	↑	n/d	(61)
Esophageal carcinoma	n/d	n/d	↑	n/d	n/d	(151, 152)
Gastric cancer	↑	n/d	↑	↓	n/d	(153–158)
Glioma	↓	n/d	n/d	n/d	n/d	(81)
Head and neck carcinoma	n/d	n/d	n/d	n/d	n/d	n/d
Liver cancer	↓	n/d	n/d	n/d	↑	(159–162)
Melanoma	n/d	n/d	↓	n/d	n/d	(163)
Nephroblastoma	n/d	n/d	n/d	n/d	n/d	n/d
Neuroblastoma	n/d	n/d	n/d	n/d	n/d	n/d
Non-small cell lung cancer	n/d	↑/↓	n/d	↑	↑/↓	(164–169)
Oral squamous cell carcinoma	↓ (saliva)	↑	n/d	n/d	n/d	(170, 171)
Osteosarcoma	n/d	n/d	n/d	n/d	n/d	n/d
Ovarian cancer	↑	↑	↑	↑	↑	(172–178)
Pancreatic cancer	↑	↑	↑	n/d	n/d	(179, 180)
Prostate cancer	↑	↑/↓	↑/↓	↑	n/d	(181–189)
Renal cell carcinoma	↓	n/d	n/d	↓	↑	(190–192)
Small cell lung carcinoma	n/d	n/d	n/d	↑	n/d	(193)
Thyroid cancer	n/d	n/d	n/d	n/d	n/d	n/d

↓ - decreased level; ↑ - increased level; n/d – no data. Arrows refer to serum miRNA level unless otherwise indicated.

MiRNAs may be loaded into small vesicles, including exosomes. Exosomal miRNAs are distributed to body fluids where they play important role in the pathogenesis of cancer by regulating all hallmarks of cancer (197). The level of exosomal miR-200 family members may serve as prognostic or diagnostic markers in various tumor types. The level of exosomal miR-200 is upregulated in ovarian cancer patients’ serum (198) and in pleural effusion of patients diagnosed with lung adenocarcinoma (199). Moreover, the level of exosomal miR-200 correlates with the invasiveness of ovarian cancer (200). Exosomal miR-200b and miR-200c may serve as independent prognostic factors in pancreatic ductal adenocarcinoma as they correlate with overall survival of patients (180). Lower expression of exosomal miR-200c and miR-141 is associated with longer overall survival of colon cancer patients (201). Moreover, miR-200 family members may play role as biomarker of cholangiocarcinoma, in particular miR-200a/c correlates with tumor stage (144).

### miRNAs as diagnostic and prognostic biomarkers

miRNAs may not only serve as diagnostic biomarkers that enable to distinguish between cancer and healthy tissue but also their levels correlate with clinical parameters and thus may have potential prognostic value (202). The combination of miRNAs may discriminate cancerous from healthy tissues with high accuracy (203, 204). The meta-analysis shows that miR-200c expression has a moderate diagnostic value in gastric cancer (205) and miR-141 may be a diagnostic marker of colorectal cancer (206). Notably, a meta-analysis of 58 articles with 8107 cancer patients revealed that in general higher expression of miR-200 family members is associated with worse survival (207, 208). Moreover, miR-200a expression was associated with unfavorable prognosis in breast cancer patients, and miR-429 correlated with shorter survival in liver cancer patients (207). Higher miR-200c expression was correlated with shorter overall survival in gastric cancer and non-small cell lung carcinoma (205, 209). However, in another meta-analysis, the expression of miR-200 family members was associated with a better prognosis in bladder cancer as it correlated with longer overall survival, recurrence-free survival, and cancer-specific survival (210). Moreover, miR-200c correlated with a better prognosis in ovarian cancer (211). Notably, it was found that serum miR-200c may be used as a marker of colorectal cancer tumor recurrence after surgery (147). Similarly, urine miR-200a was identified as marker the recurrence of non-muscle-invasive bladder cancer (137). In addition to miRNAs in the body fluids, it was found that the level of miR-141 in prostate cancer tissue may be associated with the tumor recurrence (212). Thus, more prospective clinical trials are required to determine the utility of miR-200 family members as biomarkers.

## Hallmarks of cancer

**Figure 2:**
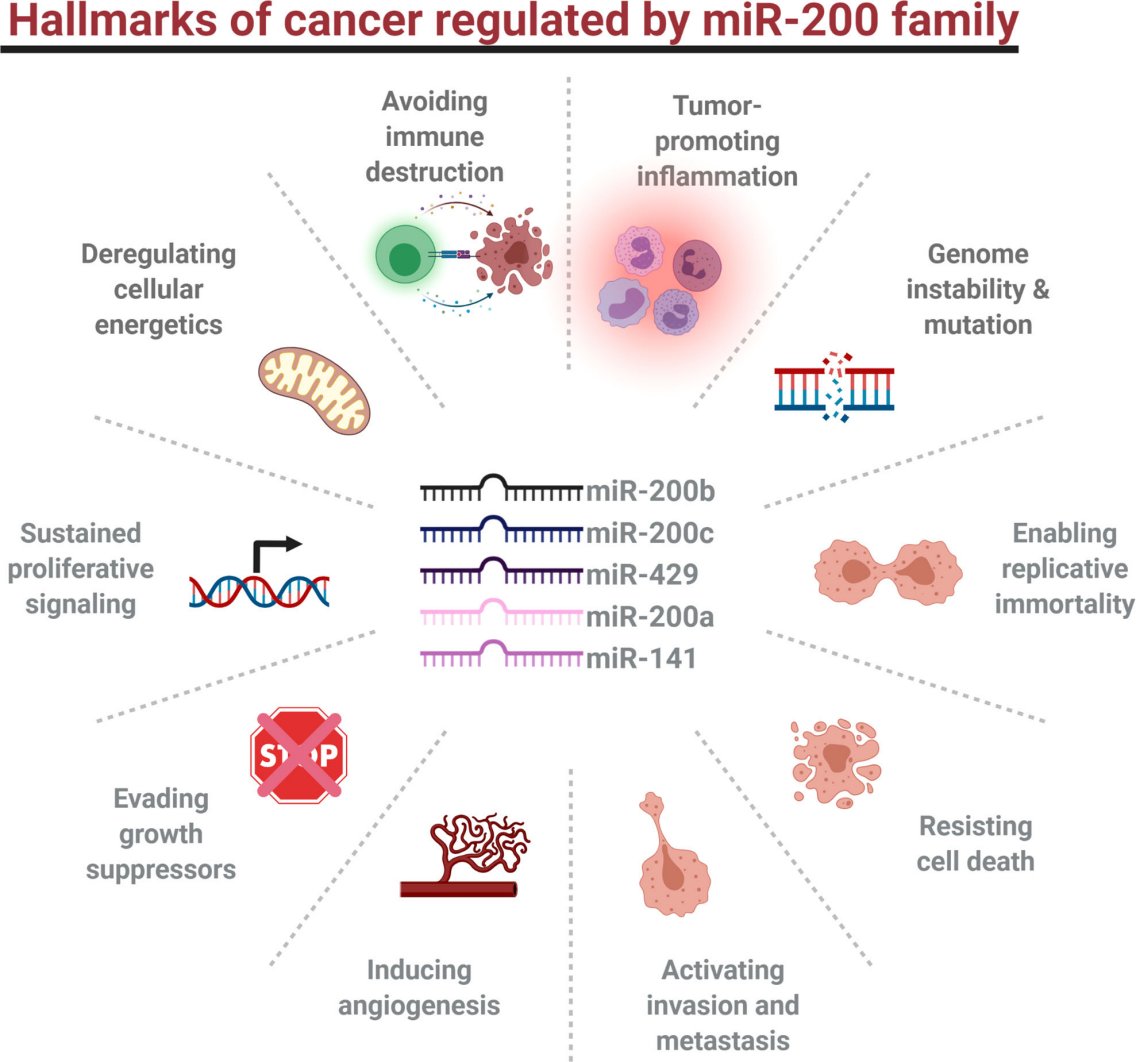
Hallmarks of cancer regulated by miR-200 family members.

Hallmarks of cancer have been first described in 2000 (213). In the updated version of their review, 14 features of human tumor development were proposed: sustaining proliferative signaling, evading tumor growth suppressors, enabling replicative immortality, activating invasion and metastasis, resisting cell death, inducing angiogenesis, avoiding immune destruction, tumor-promoting inflammation, genome instability and mutation, deregulating cellular energetics, unlocking phenotypic plasticity, polymorphic microbiomes, nonmutational epigenetic reprogramming, and senescence (214). All of them are regulated by miRNAs, including miR-200 family members (**Figure 2**) (215, 216).

## Regulation of cell proliferation by miR-200 family

The proliferation of cancer cells is one of the most important hallmarks of cancer. It is sustained by growth factors, constitutive activation of oncogenic signaling pathways, evading growth repressors like p53 or RB, and enabled replicative immortality (217). Proliferation is regulated by numerous pathways including PI3K/AKT, MEK/ERK, JAK/STAT, and β-Catenin/Wnt signaling, and by cell cycle regulators such as cyclin-dependent kinases (CDKs). The members of those pathways are regulated by miRNAs that may either promote or inhibit cancer cell proliferation. MiR-200 family has different effects on tumor cell proliferation *in vitro* depending on the cancer type (**Table 3**). Based on our literature review, miR-200a increases the proliferation of 5 cancer types and inhibits the proliferation of 13 cancer types, and there are inconsistent results concerning colorectal cancer, non-small cell lung cancer, and renal cell carcinoma. *In vivo*, miR-200a inhibits tumor growth of gastric cancer, glioma, melanoma, neuroblastoma, and prostate cancer. Furthermore, miR-200b stimulates the proliferation of 4 cancer types, and inhibits 13 cancer types, and there are inconsistent results concerning colorectal and non-small cell lung, and ovarian cell carcinoma. *In vivo* studies show miR-200b increases tumor growth of cervical and ovarian cancer and decreases tumor growth of breast cancer, glioma, head and neck cancer, non-small lung cancer, prostate cancer, and thyroid cancer. MiR-200c acts as oncomiR in 3 cancer types and as tumor suppressor miR in 13 cancer types and there are inconsistent results concerning colorectal, endometrial, and renal cell carcinoma. It decreases tumor growth *in vivo* of 9 cancer types *in vivo* and increases tumor growth of head and neck carcinoma. Moreover, miR-141 increases the proliferation of 6 cancer types, and decreases in 12 cancer types, and there are discrepancies regarding the role of miR-141 in the regulation of colorectal, non-small cell lung, and ovarian carcinoma cell proliferation. *In vivo*, miR-141 acts mainly as tumor suppressor miR and inhibits the tumor growth of numerous cancer types including colorectal, gastric, and pancreatic cancer. miR-141 increases the tumor growth of cervical and non-small cell lung cancer. Furthermore, it increases the proliferation of 5 and decreases the proliferation of 14 cancer types. *In vivo*, miR-429 inhibits tumor growth of 7 cancer types and increases tumor growth of bladder cancer only. Based on that information, miR-200 family members are suppressive miRNAs in most cancer types, however, they might also stimulate the proliferation of some types of cancer cells.

**Table 3:** The role of the miR-200 family in proliferation and tumor growth.

miRNAs	Cancer	Target	Cell proliferation *in vitro*	Tumor growth *in vivo*	Ref.
miR-200a	Acute myeloid leu-kemia	n/d	n/d	n/d	n/d
	Acute lymphoblastic leukemia	n/d	n/d	n/d	n/d
	Bladder cancer	STAT4	↓	n/d	(218)
	Breast cancer	TFAM	↓	n/d	(219)
	Cervical cancer	EGLN1	↑	n/d	(220)
	Cholangiocarcinoma	n/d	↓	n/d	(52)
	Colorectal cancer	FOXA1, RASSF2	↓/↑	n/d	(56, 221, 222)
	Endometrial cancer	PTEN	↑	n/d	(60, 223)
	Esophageal carcinoma	CTNNB1, CDH1, APC, PTEN, CTNNA1, CRMP1 and SOD2	↑	n/d	(70, 224)
	Gastric cancer	n/d	↓	↓	(225)
	Glioma	FOXA1, SIM2-s	↓	↓	(83, 226)
	Head and neck carcinoma	CD47	↓	n/d	(86)
	Liver cancer	STAT4, FOXA2	↓	n/d	(161, 162, 227)
	Melanoma	CDK6, GOLM1	↓	↓	(93, 228)
	Nephroblastoma	n/d	n/d	n/d	n/d
	Neuroblastoma	AP-2γ	↓	↓	(99)
	Non-small cell lung cancer	RHPN2	↑/↓	n/d	(229, 230)
	Oral squamous cell carcinoma	n/d	n/d	n/d	n/d
	Osteosarcoma	ZEB1	↓	n/d	(104)
	Ovarian cancer	PDCH9	↑	n/d	(111, 113)
	Pancreatic cancer	DEK	↓	n/d	(231, 232)
	Prostate cancer	BRD4	↓	↓	(233, 234)
	Renal cell carcinoma	CBL, SIRT1, SPAG9, TGFβ2	↓/↑	n/d	(124, 126, 127, 235, 236)
	Small cell lung carcinoma	n/d	n/d	n/d	n/d
	Thyroid cancer	FOXA1	↓	n/d	(130)
miR-200b	Acute myeloid leu-kemia	n/d	↓	n/d	(237)
	Acute lymphoblastic leukemia	n/d	n/d	n/d	n/d
	Bladder cancer	FSCN1	↓	n/d	(238)
	Breast cancer	IKBKB, FUT4, radixin, SP1	↓	↓	(239–242)
	Cervical cancer	FOXG1	↑	↑	(243)
	Cholangiocarcinoma	n/d	↑	n/d	(244)
	Colorectal cancer	RECK, TUBB3, Wnt1	↑/↓	n/d	(245–247)
	Endometrial cancer	PTEN	↑	n/d	(60, 223)
	Esophageal carcinoma	n/d	n/d	n/d	n/d
	Gastric cancer	ZEB2	↓	n/d	(248)
	Glioma	CD133, CREB1, ERK5	↓	↓	(249–251)
	Head and neck carcinoma	Notch1	↓	↓	(252)
	Liver cancer	HMGB3	↓	n/d	(90)
	Melanoma	n/d	↓	n/d	(253)
	Nephroblastoma	IKK-β	↓	n/d	(98)
	Neuroblastoma	n/d	n/d	n/d	n/d
	Non-small cell lung cancer	ABCA1, p70S6K1, RhoE	↑/↓	↓	(254–256)
	Oral squamous cell carcinoma	Kindlin-2, ZEB2	↓	n/d	(257)
	Osteosarcoma	ZEB1	↓	n/d	
	Ovarian cancer	ATAD2, ING5	↑/↓	↑	(258, 259)
	Pancreatic cancer	n/d	n/d	n/d	n/d
	Prostate cancer	n/d	↓	↓	(233, 260–262)
	Renal cell carcinoma	n/d	↑	n/d	(124)
	Small cell lung carcinoma	n/d	n/d	n/d	n/d
	Thyroid cancer	RAP1B	↓	↓	(132)
miR-200c	Acute myeloid leu-kemia	n/d	n/d	n/d	n/d
	Acute lymphoblastic leukemia	n/d	n/d	n/d	n/d
	Bladder cancer	BMI-1, E2F3, LDHA	↓	n/d	(36, 263)
	Breast cancer	BMI-1, KRAS, PDE7B, XIAP	↓	↓	(264–269)
	Cervical cancer	MAP4K4	↓	n/d	(270)
	Cholangiocarcinoma	n/d	n/d	n/d	n/d
	Colorectal cancer	CDK2, RASSF2	↑/↓	n/d	(56, 271)
	Endometrial cancer	PTEN, PTENP1, MALAT1	↑/↓	↓	(60, 272; 273)
	Esophageal carcinoma	n/d	n/d	n/d	n/d
	Gastric cancer	EDNRA, FN1	↓	n/d	(274, 275)
	Glioma	MSN	↓	↓	(79)
	Head and neck carcinoma	PTEN	↑	↑	(276)
	Liver cancer	MAD2L1	↓	n/d	(88)
	Melanoma	n/d	↓	↓	(277)
	Nephroblastoma	FRS2, IKK-β	↓	n/d	(97, 98, 278)
	Neuroblastoma	n/d	n/d	n/d	n/d
	Non-small cell lung cancer	LDHA	↓	↓	(279, 280)
	Oral squamous cell carcinoma	n/d	↓	n/d	(102)
	Osteosarcoma	AKT2	↓	↓	(105)
	Ovarian cancer	n/d	↑	↓	(281, 282)
	Pancreatic cancer	n/d	↑	n/d	(283)
	Prostate cancer	AMACR, ZEB2	↓	↓	(122, 284, 285)
	Renal cell carcinoma	SLC6A1	↑/↓	n/d	(124, 128)
	Small cell lung carcinoma	n/d	n/d	n/d	n/d
	Thyroid cancer	RAP1B	↓	↓	(132)
miR-141	Acute myeloid leu-kemia	RAB32	↓	↓	(286)
	Acute lymphoblastic leukemia	TRAF5	↓	n/d	(32)
	Bladder cancer	n/d	n/d	n/d	n/d
	Breast cancer	ANP32E, HMGB1	↓	↓	(287, 288)
	Cervical cancer	FOXA2	↑	↑	(289)
	Cholangiocarcinoma	n/d	↑	n/d	(244)
	Colorectal cancer	PHLPP2, RASSF2	↑/↓	↓	(56, 58, 290, 291)
	Endometrial cancer	n/d	↑	n/d	(60)
	Esophageal carcinoma	n/d	↓	n/d	(67)
	Gastric cancer	YAP1, TAZ	↓	↓	(75, 292)
	Glioma	SKA2	↓	↓	(77)
	Head and neck carcinoma	EGFR	↓	↓	(85)
	Liver cancer	TGFβR1	↓	n/d	(91)
	Melanoma	n/d	n/d	n/d	n/d
	Nephroblastoma	n/d	n/d	n/d	n/d
	Neuroblastoma	FUS	↓	↓	(293)
	Non-small cell lung cancer	HOXC13, KLF9	↑/↓	↑	(193, 294, 295)
	Oral squamous cell carcinoma	n/d	n/d	n/d	n/d
	Osteosarcoma	GLI2	↓	n/d	(106, 296)
	Ovarian cancer	n/d	↑/↓	n/d	(112, 297)
	Pancreatic cancer	MAP4K4	↓	↓	(298)
	Prostate cancer	KLF9, RUNX1	↑	n/d	(299, 300)
	Renal cell carcinoma	n/d	↑	n/d	(124)
	Small cell lung carcinoma	n/d	n/d	n/d	n/d
	Thyroid cancer	IRS2	↓	↓	(129)
miR-429	Acute myeloid leukemia	n/d	n/d	n/d	n/d
	Acute lymphoblastic leukemia	n/d	n/d	n/d	n/d
	Bladder cancer	CDKN2B	↑	↑	(39)
	Breast cancer	FN1	↓	n/d	(301)
	Cervical cancer	IKK-β, ZEB1	↓	↓	(46, 302)
	Cholangiocarcinoma	n/d	n/d	n/d	n/d
	Colorectal cancer	HMGB3, Onecut2	↓	↓	(55, 59)
	Endometrial cancer	n/d	↑	n/d	(60)
	Esophageal carcinoma	Bcl-2, SP1, Slug, RAB23	↓	↓	(65, 68, 69)
	Gastric cancer	FSCN1, c-MYC	↓	↓	(71, 74, 303)
	Glioma	SOX2	↓	n/d	(82)
	Head and neck carcinoma	n/d	↓	n/d	(304)
	Liver cancer	RAB23	↓	n/d	(305)
	Melanoma	AKT1	n/d	↓	(306)
	Nephroblastoma	c-MYC, IKK-β	↓	n/d	(96, 98)
	Neuroblastoma	IKK-β	↓	↓	(307)
	Non-small cell lung cancer	DLC−1, PTEN, RASSF8, TIMP2	↑	n/d	(308, 309)
	Oral squamous cell carcinoma	ZEB1	↓	n/d	(310)
	Osteosarcoma	ZEB1, HOXA9	↓	n/d	(103, 107)
	Ovarian cancer	n/d	n/d	n/d	n/d
	Pancreatic cancer	NT-3	↓	n/d	(117)
	Prostate cancer	p27Kip1	↑	n/d	(311)
	Renal cell carcinoma	AKT1	↑	↓	(124, 312)
	Small cell lung carcinoma	n/d	n/d	n/d	n/d
	Thyroid cancer	ZEB1	↓	n/d	(131)

↓ - suppression by miRNA; ↑ - promotion by miRNA; n/d – no data.

MiR-200 family members exert their effects by targeting several mRNAs associated with cancer cell proliferation. MiR-200a targets Cyclin-Dependent Kinase 6 (CDK6) in melanoma and thus causes cell-cycle arrest and decreases cancer cell proliferation (93). MiR-429 targets p27Kip1, an inhibitor of the cell cycle, and its overexpression promotes the proliferation of prostate cancer cells (311). Furthermore, the miR-200 family targets the members of key pathways regulating cancer cell proliferation. PI3K/AKT pathway promotes proliferation, migration, invasion, and EMT (epithelial-mesenchymal transition) (313). AKT1/2 is targeted by miR-200c in osteosarcoma (105), and by miR-429 in melanoma (306) and renal cell carcinoma (312). Downregulation of AKT1/2 by miRNAs inhibits cancer cell proliferation *in vitro* and tumor growth *in vivo*. Moreover, the miR-200 family targets PTEN, a key suppressor of the PI3K/AKT pathway (314). It is targeted by miR-200a in endometrial cancer (223) and esophageal carcinoma (70), miR-200b in endometrial cancer (223), miR-200c in endometrial cancer (273) and head and neck carcinoma (276), and miR-429 in non-small cell lung cancer (308) resulting in increased of cancer cells proliferation *in vitro*.

The second most important pathway regulating cell proliferation is MEK/ERK pathway. KRAS which activates MEK/ERK is targeted by miR-200c in breast cancer. MiR-200c thus has an inhibitory effect on breast cancer cell proliferation and tumor growth (265). Moreover, miR-200b targets p70S6K1 and inhibits lung cancer cell proliferation *in vitro* and tumor growth *in vivo* (256). Whereas, miR-429 targets RAB23 in esophageal and liver carcinomas resulting in the suppression of cell proliferation, migration, and invasion (69, 305). On the contrary, MEK/ERK inhibitor Ras Association Domain Family Member 2 (RASSF2) is targeted by miR-200a, miR-200c, miR-141 in colorectal cancer, and Ras Association Domain-Containing Protein 8 (RASSF8) by miR-429 in non-small lung carcinoma which results in increased proliferation (56, 308). Similarly, other signaling pathways regulating cell proliferation are targeted by miRNAs. MiR-200a binds STAT4 in bladder and liver cancer and decreases cell proliferation *in vitro* (162, 218). In esophageal carcinoma, however, miR-200a acts as oncomiR and targets APC, an inhibitor of β-Catenin/Wnt signaling (70).

## Regulation of cell migration and invasiveness by miR-200 family

Cancer cell migration and invasion are multistep processes that involve cytoskeleton remodeling, interaction with extracellular matrix (ECM), and then its digestion. All these steps are regulated by miRNAs that suppress or promote cancer progression (7).

In this study, we summarized the knowledge about miR-200 family regulation of cancer cell migration and invasion *in vitro* (**Table 4**). MiR-200 family members suppress cell migration and invasiveness in most types of cancer. MiR-200a increases the migration of 2 cancer types and the invasion of 3 cancer types and it decreases the migration of 12 and invasion of 13 cancer types. MiR-200b increases the migration of renal cell cancer and decreases the migration of 16 cancer types and invasion of 15 cancer types. Furthermore, miR-200c promotes the migration and invasion of head and neck carcinoma. On the other hand, miR-200c inhibits the migration of 15 and invasion of 13 types of cancers. MiR-141 decreases the migration of 13 types of cancer cells and invasion of 13 cancer types. However, it increases the migration of bladder and renal cell carcinoma, and it stimulates the invasion of bladder, cervical, and non-small lung cancer. Likewise, miR-429 decreases the migration and invasion of 15 cancer types, while it increases the migration of renal cell carcinoma and migration and invasion of non-small lung carcinoma.

**Table 4:** Members of the miR-200 family regulating cancer cell migration and invasion.

miRNAs	Cancer	Target	Cell migration *in vitro*	Cell invasion *in vitro*	Ref.
miR-200a	Acute myeloid leukemia	n/d	n/d	n/d	n/d
	Acute lymphoblastic leukemia	n/d	n/d	n/d	n/d
	Bladder cancer	DICER	n/d	↑	(41)
	Breast cancer	ELK3, EPHA2	↓	↓	(315, 316)
	Cervical cancer	n/d	n/d	n/d	n/d
	Cholangiocarcinoma	n/d	n/d	↓	(52)
	Colorectal cancer	FOXA1	↓	↓	(221, 222)
	Endometrial cancer	n/d	n/d	n/d	n/d
	Esophageal carcinoma	CTNNB1, CDH1, APC, PTEN, CTNNA1 and SOD2	↑	↑	(70)
	Gastric cancer	n/d	↓	↓	(225)
	Glioma	FOXA1, SIM2-s	↓	↓	(83, 226)
	Head and neck carcinoma	CD47	↓	↓	(86)
	Liver cancer	STAT4, Foxa2	↓	↓	(161, 162, 227)
	Melanoma	GOLM1	↓	↓	(228)
	Nephroblastoma	n/d	n/d	n/d	n/d
	Neuroblastoma	n/d	n/d	n/d	n/d
	Non-small cell lung cancer	HGF	↓	↓	(100)
	Oral squamous cell carcinoma	n/d	n/d	n/d	n/d
	Osteosarcoma	ZEB1	↓	n/d	(104)
	Ovarian cancer	PDCH9	↑	↑	(111, 113)
	Pancreatic cancer	DEK	↓	↓	(231, 232, 317)
	Prostate cancer	n/d	n/d	↓	(233)
	Renal cell carcinoma	TGFβ2	↓	↓	(235)
	Small cell lung carcinoma	n/d	n/d	n/d	n/d
	Thyroid cancer	FOXA1	↓	↓	(130)
miR-200b	Acute myeloid leukemia	n/d	n/d	↓	(237)
	Acute lymphoblastic leukemia	n/d	n/d	n/d	n/d
	Bladder cancer	FSCN1	↓	↓	(238)
	Breast cancer	IKBKB, FUT4, PKCα, radixin,	↓	↓	(240–242, 318, 319)
	Cervical cancer	FoxG1, RhoE	↓/↑	↓/↑	(45, 243, 320)
	Cholangiocarcinoma	SUZ12 and ROCK2	↓	↓	(51)
	Colorectal cancer	TUBB3	↓	↓	(247)
	Endometrial cancer	TIMP2	↓	n/d	(321)
	Esophageal carcinoma	Kindlin-2	↓	↓	(322)
	Gastric cancer	ZEB2	↓	↓	(248)
	Glioma	CD133, ERK5	↓	↓	(250, 251)
	Head and neck carcinoma	Notch1	↓	↓	(252)
	Liver cancer	HMGB3	↓	n/d	(90)
	Melanoma	n/d	↓	↓	(253)
	Nephroblastoma	IKK-β	↓	↓	(98)
	Neuroblastoma	n/d	n/d	n/d	n/d
	Non-small cell lung cancer	ABCA1, p70S6K1, RhoE, TIF1γ	↑/↓	↑/↓	(255, 256, 323, 324)
	Oral squamous cell carcinoma	Kindlin-2, ZEB2	↓	↓	(257)
	Osteosarcoma	ZEB1	↓	↓	
	Ovarian cancer	n/d	n/d	n/d	n/d
	Pancreatic cancer	n/d	n/d	n/d	n/d
	Prostate cancer	n/d	↓	↓	(233, 261, 262)
	Renal cell carcinoma	n/d	↑	n/d	(124)
	Small cell lung carcinoma	n/d	n/d	n/d	n/d
	Thyroid cancer	RAP1B	↓	↓	(132, 325)
miR-200c	Acute myeloid leukemia	n/d	n/d	n/d	n/d
	Acute lymphoblastic leukemia	n/d	n/d	n/d	n/d
	Bladder cancer	BMI-1, E2F3, LDHA, RECK	↑/↓	↑/↓	(36, 38, 263)
	Breast cancer	n/d	↓	↓	(267, 268, 326)
	Cervical cancer	MAP4K4	↓	n/d	(270)
	Cholangiocarcinoma	SUZ12 and ROCK2	↓	↓	(51)
	Colorectal cancer	n/d	↓	↓	(327)
	Endometrial cancer	PTEN, PTENP1	↑/↓	↑/↓	(272, 328; 273)
	Esophageal carcinoma	n/d	n/d	n/d	n/d
	Gastric cancer	EDNRA, FN1	↓	↓	(274, 275)
	Glioma	MSN	↓	↓	(79)
	Head and neck carcinoma	PTEN	↑	↑	(276)
	Liver cancer	MAD2L1	↓	↓	(88)
	Melanoma	n/d	↓	n/d	(277)
	Nephroblastoma	FRS2, IKK-β	↓	↓	(97, 98)
	Neuroblastoma	n/d	n/d	n/d	n/d
	Non-small cell lung cancer	LDHA	↓	n/d	(280)
	Oral squamous cell carcinoma	ZEB1	↓	↓	(329)
	Osteosarcoma	AKT2	↓	n/d	(105)
	Ovarian cancer	n/d	↓	↓	(281, 282)
	Pancreatic cancer	n/d	n/d	↓	(283, 330)
	Prostate cancer	AMACR, ZEB2	↓	↓	(122, 284, 285)
	Renal cell carcinoma	SLC6A1	↑/↓	↓	(124, 128)
	Small cell lung carcinoma	n/d	n/d	n/d	n/d
	Thyroid cancer	RAP1B	↓	↓	(132)
miR-141	Acute myeloid leukemia	n/d	n/d	n/d	n/d
	Acute lymphoblastic leukemia	n/d	n/d	n/d	n/d
	Bladder cancer	n/d	n/d	n/d	n/d
	Breast cancer	ANP32E, ZEB1/2	↓	↓	(287, 331)
	Cervical cancer	FOXA2	n/d	↑	(289)
	Cholangiocarcinoma	n/d	n/d	n/d	n/d
	Colorectal cancer	EGFR	↓	↓	(332)
	Endometrial cancer	n/d	n/d	n/d	n/d
	Esophageal carcinoma	n/d	↓	↓	(67)
	Gastric cancer	TAZ	↓	↓	(292)
	Glioma	SKA2	↓	↓	(77)
	Head and neck carcinoma	EGFR	↓	↓	(85)
	Liver cancer	TGFβR1	↓	↓	(91)
	Melanoma	n/d	n/d	n/d	n/d
	Nephroblastoma	n/d	n/d	n/d	n/d
	Neuroblastoma	FUS	↓	↓	(293)
	Non-small cell lung cancer	KLF9	n/d	↑	(295)
	Oral squamous cell carcinoma	n/d	n/d	n/d	n/d
	Osteosarcoma	FUS	↓	↓	(296)
	Ovarian cancer	n/d	↓	↓	(297)
	Pancreatic cancer	MAP4K4, TM4SF1	↓	↓	(298, 333)
	Prostate cancer	RUNX1	↓	↓	(300)
	Renal cell carcinoma	n/d	↑	n/d	(124)
	Small cell lung carcinoma	n/d	n/d	n/d	n/d
	Thyroid cancer	IRS2	↓	↓	(129)
miR-429	Acute myeloid leukemia	n/d	n/d	n/d	n/d
	Acute lymphoblastic leukemia	n/d	n/d	n/d	n/d
	Bladder cancer	n/d	↓	↓	(334)
	Breast cancer	FN1	↓	↓	(301)
	Cervical cancer	ZEB1	↓	↓	(302)
	Cholangiocarcinoma	n/d	n/d	n/d	n/d
	Colorectal cancer	Onecut2, PAK6	↓	↓	(55, 335)
	Endometrial cancer	n/d	n/d	n/d	n/d
	Esophageal carcinoma	Bcl-2, Slug, SP1	↓	↓	(65, 68)
	Gastric cancer	Sp1, Notch1	↓	↓	(336, 337)
	Glioma	BMK1, SOX2	↓	↓	(82, 338)
	Head and neck carcinoma	n/d	↓	↓	(304)
	Liver cancer	CRKL, RAB23	↓	↓	(89, 305)
	Melanoma	LIMK1	↓	↓	(94)
	Nephroblastoma	IKK-β	↓	↓	(98)
	Neuroblastoma	IKKβ	↓	↓	(307)
	Non-small cell lung cancer	PTEN, RASSF8, TIF1γ and TIMP2,	↑	↑	(308, 323)
	Oral squamous cell carcinoma	n/d	n/d	n/d	n/d
	Osteosarcoma	ZEB1, HOXA9	↓	↓	(103, 107)
	Ovarian cancer	p15PAF	↓	n/d	(339)
	Pancreatic cancer	NT-3	n/d	↓	(117)
	Prostate cancer	n/d	n/d	n/d	n/d
	Renal cell carcinoma	n/d	↑	n/d	(124)
	Small cell lung carcinoma	n/d	n/d	n/d	n/d
	Thyroid cancer	ZEB1	↓	↓	(131)

↓ - suppression by miRNA; ↑ - promotion by miRNA; n/d – no data.

The epithelial-mesenchymal process (EMT) is one of the crucial cellular processes in cancer cell invasion. During this process, epithelial cells gain mesenchymal capabilities including migration. The regulation of EMT is complex and is modulated by numerous signaling pathways including TGFβ and Wnt/β-catenin and transcription factors such as Snail, Slug, ZEB1/2, and TWIST1/2 (7, 340). MiR-200 family is a well-known regulator of EMT because of targeting ZEB1 and ZEB2 (341) which are key inducers of this process (342). ZEB1/2 is targeted by miR-200a [osteosarcoma (104)], 200b [gastric cancer, oral squamous cell carcinoma, osteosarcoma (248, 257)], miR-200c [oral squamous cell carcinoma, prostate cancer (285, 329)], miR-141 [breast cancer (331)], and miR-429 [cervical cancer, osteosarcoma, thyroid cancer (103, 131, 302)]. Moreover, the miR-200 family targets other regulators of EMT. MiR-200a targets TGFβ2 in renal cell carcinoma and inhibits the invasion and migration of cancer cells (235). MiR-141 suppresses liver cancer cell invasion and migration by targeting TGFβR1 (91). Moreover, miR-429 targets Slug, a transcription factor that belongs to the Snail family and activates EMT. Thus, miR-429 inhibits the migration and invasion of esophageal cancer cells (68). On the contrary, miR-200a targets Catenin Beta 1 (CTNNB1), the member of the Wnt/β-catenin pathway, and promotes the invasion and migration of esophageal cancer cells (70).

Cytoskeleton remodeling is a vital step in cells motility. It leads to the formation of cell membrane protrusions, then to cell contraction and retraction of rear-end (343). Moesin and radixin belong to ERM (ezrin-radixin-moesin) protein family that is responsible for cytoskeleton structure and cell migration (344). MiR-200c targets moesin in glioma (79) and miR-200b targets radixin in breast cancer cells (242). By targeting them, miR-200b and miR-200c suppress cancer progression (70). Fascin 1 (FSCN1) is an actin-bundling protein involved in the formation of protrusions. It is targeted by miR-200b leading to the decrease of cancer cell migration and invasion in bladder cancer (238). RhoE is a member of Ras superfamily and is responsible for actin reorganization. Both, miR-200b and miR-200c are described to target RhoE and inhibit the invasion of non−small cell lung cancer cells (344). Kindlin-2 is another protein involved in extracellular matrix regulation. It is reported to be directly targeted by miR-200b in esophageal carcinoma and in oral squamous cell carcinoma which causes the inhibition of cancer cell migration and invasion (79). TUBB3 (Tubulin Beta 3 Class III), a main cytoskeletal microtubule protein engaged in various processes including cancer invasion and migration, is targeted by miR-200b in colorectal cancer leading to the suppression of migration and invasion of cancer cells (247).

The ECM proteolysis and interactions with the cancer cell cytoskeleton play a crucial role in the migration and invasion of tumor cells (345). MiRNA-200c targets fibronectin (FN1), the component of the extracellular matrix, in gastric cancer cells and acts as a suppressor of invasion and migration (274). On the contrary, miR-200 family members also promote cancer progression by targeting the inhibitors of metalloproteinases that digest ECM. miR-200c targets RECK in bladder cancer (38), whereas TIMP2 is targeted by miR-200b in endometrial cancer cells (66, 257). Moreover, miR-200a regulates cell-to-cell adhesion by targeting CDH1 (cadherin 1) (70).

## Regulation of apoptosis by miR-200 family

Apoptosis is so-called programmed cell death, essential for maintaining tissue homeostasis. The mechanism of apoptosis is complex and involves several steps. Each of these steps can be impaired, thus leading to carcinogenesis (346). The major role in apoptosis belongs to caspases, which may be both initiators and executioners of apoptosis (347). Numerous studies have reported the influence of miRNAs on the regulation of both the intrinsic and the extrinsic apoptosis pathways (348).

The miR-200 family members act mainly as apoptosis promoters (**Table 5**). miR-200a promotes apoptosis in 6 cancer types and inhibits apoptosis only in cervical cancer. There are discordant results concerning the role of miR-200a in the regulation of non-small cell lung cancer. MiR-200b promotes apoptosis in 5 cancer types and miR-200c promotes apoptosis in 6 cancer types. miR-141 promotes apoptosis in 6 cancer types, and inhibits apoptosis in pancreatic cancer, but its role in prostate cancer is inconsistent. miR-429 promotes apoptosis in 9 cancer types and inhibits apoptosis only in colorectal cancer.

**Table 5:** Members of the miR-200 family regulating apoptosis.

miRNAs	Cancer	Target	Apoptosis *in vitro*	Ref.
miR-200a	Cervical cancer	EGLN1	↓	(220)
	Gastric cancer	A20	↑	(349)
	Liver cancer	n/d	↑	(227)
	Nephroblastoma	CDC7	↑	(350)
	Non-small cell lung cancer	HGF, RHPN2	↓/↑	(100, 351)
	Prostate cancer	BRD4, SIRT1, SPAG9	↑	(126, 234, 236)
	Renal cell carcinoma	CBL, SPAG9	↑	(126, 127)
	Thyroid cancer	FOXA1	↑	(130)
miR-200b	Acute myeloid leukemia	n/d	↑	(237)
	Breast cancer	Sp1	↑	(239)
	Colorectal cancer	TUBB3, Wnt1	↑	(246, 247)
	Oral squamous cell carcinoma	CDK2, PAF	↑	(352)
	Ovarian cancer	ATAD2	↑	(259)
miR-200c	Breast cancer	XIAP	↑	(264)
	Colorectal cancer	CDK2	↑	(271)
	Gastric cancer	EDNRA	↑	(275)
	Liver carcinoma	MAD2L1	↑	(88)
	Nephroblastoma	n/d	↑	(278)
	Non-small cell lung cancer	n/d	↑	(279)
miR-141	Acute lymphoblastic leukemia	TRAF5	↑	(32)
	Colorectal cancer	EGFR	↑	(332)
	Gastric cancer	YAP1	↑	(75)
	Head and neck carcinoma	EGFR	↑	(85)
	Osteosarcoma	GLI2	↑	(106)
	Pancreatic cancer	MAP4K4	↓	(298)
	Prostate cancer	KLF9	↓/↑	(299, 300)
	Thyroid cancer	IRS2	↑	(129)
miR-429	Gastric carcinoma	Bcl-2	↑	(303, 353)
	Glioblastoma	Bcl-2	↑	
	Nephroblastoma	c-MYC	↑	(96)
	Colorectal cancer	HMGB3	↑	(59)
	Osteosarcoma	ZEB1	↑	(103)
	Glioblastoma	SOX2	↑	(82)
	Esophageal carcinoma	Bcl-2, SP1	↑	(65)
	Thyroid cancer	ZEB1	↑	(131)
	Colorectal cancer	SOX2	↓	(53)
	Cervical carcinoma	IKKb	↑	(46)

↓ - suppression by miRNA; ↑ - promotion by miRNA; n/d – no data

MiR-200a enhances TRAIL-triggered apoptosis in gastric cancer cells by targeting TNFα-induced protein 3 (A20) (349). Moreover, miR-200a shows pro-apoptotic activity in the human hepatocellular carcinoma cell line (227). In Wilms tumor cells, miR-200a promotes apoptosis by targeting CDC7 (350). Similarly, miR-200a can promote apoptosis in prostate cancer cells through BRD4/AR signaling pathway (234), by directly targeting SIRT1 (236) or by targeting Sperm-associated antigen 9 (SPAG9) (100). The last-mentioned protein, SPAG9, is an oncogene protein that regulates renal cell carcinoma progression. Accordingly, its regulation by miR-200 also has a stimulatory effect on apoptosis in renal cell carcinoma (126). Moreover, miR-200a has been shown to promote apoptosis of renal cell carcinoma cells by targeting CBL (127). Furthermore, by targeting FOXA1, miR-200a also promotes cell apoptosis in anaplastic thyroid carcinoma (130). In contrast, miR-200a suppresses apoptosis and promotes the proliferation of cervical cancer cells by targeting EGLN1 (220). In the case of NSCLC, miR-200 demonstrates a two-fold action. It promotes apoptosis by downregulation of the hepatocyte growth factor (HGF) (100).

MiR-200b has been shown to have pro-apoptotic effects among all the studied types of cancer. In breast cancer, miR-200b induces apoptosis and inhibits cell proliferation by directly targeting Sp1 (239). Moreover, high expression of miR-200b appeared to be an independent prognostic factor for patients with breast cancer (239). Moreover, miR-200b-3p shows pro-apoptotic effects in colorectal cancer by inactivating the Wnt/β-catenin signaling pathway (246). Likewise, the same occurs in esophageal squamous cell carcinoma where miR-200b also modulates the Wnt/β-catenin signaling pathway by targeting CDK2 and PAF. Therefore, by inducing cell cycle arrest and apoptosis, miR-200b alleviates cancer cell growth (246). In ovarian cancer, miR-200b significantly increases the apoptosis rate of the cancer cells by targeting ATAD2 and regulating PI3K/AKT signaling pathway (259). miR-200b inhibited cancer growth and induced cell apoptosis in the oxaliplatin-resistant colorectal cancer cells through suppression of βIII-tubulin protein expression (247).

MiR-200c also has a pro-apoptotic effect in all studied types of cancer. In triple-negative breast cancer cells, microRNA-200c was found to downregulate XIAP expression and thus suppress proliferation and promote apoptosis of cancer (264). In gastric cancer, miR-200c promotes apoptosis of the tumor cells by downregulation of endothelin receptor A (EDNRA) expression (275). Similarly, in human hepatocellular carcinoma miR-200c induces apoptosis *via* suppressing MAD2L1 (88). Overexpression of miR-200c has been proven to promote apoptosis in Wilms tumors *via* downregulation of the Akt/GLUT1 signaling pathway (278). Furthermore, the high expression of miR-200c was also reported to enhance apoptosis in lung cancer tissues, where it caused activation of the JNK signaling pathway and upregulation of an ER stress-related protein, RECK.

MiR-141 demonstrates apoptosis-promoting activity in the majority of cancer types. However, miR-141 appears to modulate apoptosis in a bidirectional manner in prostate cancer. miR-141 induces prostate cancer cell apoptosis *via* targeting Runt-related transcription factor 1 (RUNX1) (300). However, another study reported that miR141 significantly reduced cell apoptosis, thus appearing to be a novel oncogene miRNA, which promotes prostate tumorigenesis *via* suppressing a key transcription factor kruppel-like factor-9 (KLF9) (299). Therefore, it remains to define the role that miR-141 plays in the apoptosis of prostate cancer cells. In T-cell acute lymphoblastic leukemia cells the upregulation of miR-141-3p significantly decreased cancer cell proliferation and promoted its apoptosis by direct targeting tumor necrosis factor receptor-associated factor 5 (TRAF5) (32). Furthermore, among colorectal cancer cells, miR-141- has been proven to reduce cell growth and induce apoptosis and differentiation of colorectal cancer cells by targeting EGFR (332). Similarly, in head and neck squamous cell carcinoma, where miR-141, also by suppressing EGFR signaling, inhibits tumor growth, and promotes apoptosis in cancer (85). Moreover, in pancreatic cancer cells, miR-141 acts as a tumor suppressor by targeting MAP4K4, which knockdown inhibits cell proliferation and induces G1 arrest and apoptosis (298). Moreover, since insulin receptor substrate 2 (IRS2), a known oncogene, was confirmed to be a direct target of miR-141, its overexpression blocks cell proliferation and induces cell apoptosis of thyroid cancer (129).

MiR-429 acts in a pro-apoptotic manner in the majority of cancer types. In colorectal cancer, miR-429 has been demonstrated to exert a two-fold effect. By mediating high mobility group box 3 (HMGB3) miR-429 promotes apoptosis in colorectal cancer cells (59). However, directly targeting SOX2 may prevent cell death (53). Therefore, it is unclear whether miR-429 plays an oncogenic or tumor-suppressive role in colorectal cancer. Remarkably, SOX2 is also directly targeted by miR-429 in glioblastoma multiforme, but in this cancer downregulation of SOX2 inhibits cell proliferation and induces apoptosis (82). Moreover, in glioblastoma miR-429 induces apoptosis also by targeting Bcl-2. Furthermore, downregulation of Bcl-2 by miR-429 induces apoptosis in gastric carcinoma (303, 353). In both types of cancers, overexpression of miR-429 inhibits Bcl-2-mediated cell survival (303, 353). In esophageal carcinoma upregulation of miR-429, by targeting both Bcl-2 and SP1, promotes apoptosis in cancer cells (65). Another direct target for miR-429 is ZEB1 which inhibition results in the induction of apoptosis in osteosarcoma (103) and thyroid cancer cells (131). Moreover, miR-429 enhances apoptosis in nephroblastoma, by targeting c-myc (96) and also in cervical carcinoma, through the NF-κB pathway by targeting IKKβ (46).

## Regulation of angiogenesis by miR-200 family

Angiogenesis is the process of formation of new capillaries from a pre-existing vasculature, which is a crucial factor affecting tumor formation, progression, and metastasis (217). Malignant tumors create an environment that favors the predominance of proangiogenic factors over antiangiogenic factors, resulting in inappropriate vessel growth towards the neoplastic lesion. Many proangiogenic and antiangiogenic agents have been identified. They may be secreted by endothelial cells, tumor cells, or by the surrounding stroma. MiRNAs may target genes involved in angiogenesis, but on the other hand, their expression can be modulated *via* pro-angiogenic or anti-angiogenic factors (354).

miR-200 family members act both as angiogenesis inhibitors and promoters (**Table 6**). miR-200a, miR-200b and miR-429 inhibit angiogenesis. miR-200c is described as an angiogenesis inhibitor, but it promotes angiogenesis in pancreatic cancer. Similarly, miR-141 is described to inhibit angiogenesis, but it also promotes angiogenesis in small-cell lung cancer and ovarian cancer.

**Table 6:** Regulation of angiogenesis by miR-200 family members.

miRNAs	Cancer	Target	Angiogenesis	Ref.
miR-200a	n/d	n/d	↓	(355)
miR-200b	n/d	n/d	↓	(355)
	Prostate cancer	n/d	↓	(261)
miR-200c	n/d	n/d	↓	(355)
	Pancreatic cancer	n/d	↑	(356)
miR-141	Small cell lung cancer	KLF12	↑	(193)
	Ovarian cancer	n/d	↑	(357)
	n/d	CXCL12β, GAB1, GATA6, NRP1, TGFβ2	↓	(355)
miR-429	n/d	n/d	↓	(355)

↓ - suppression by miRNA; ↑ - promotion by miRNA; n/d – no data.

In 2011, Chan et al. first described the effect of miR-200b on the suppression of angiogenesis, thus identifying the first member of the miR-200 family to have an inhibitory effect on this process (358). Several years after this finding, it was confirmed that all members of the miR-200 family suppress angiogenesis (193). In prostate cancer cells, miR-200b reverses the angiogenic switch (261). Nevertheless, some members of the miR-200 family, more specifically miR-200c and miR-141, may demonstrate pro-angiogenic effects as well. For instance, increased expression of miR-200c in pancreatic cancer endothelial cells is observed and its inhibition significantly reduces cancer cell migration and angiogenesis, confirming the pro-angiogenic effects of miR-200c in pancreatic cancer (356). MiR-141 is another member of the miR-200 family that, in addition to its anti-angiogenic effects (355), also demonstrates angiogenesis-promoting activity in ovarian cancer. The ovarian cancer-secreted miR141-3p promotes endothelial cell angiogenesis by activating the JAK/STAT3 and NF-κB signaling pathways (357). Likewise, in small cell lung cancer (SCLC), miR-141 promotes angiogenesis *via* the KLF12 pathway (193).

## Regulation of drug resistance by miR-200 family

Drug resistance in cancer cells, resulting in reduced or no response to the administered therapy and poorer overall survival of cancer patients is a limiting factor for this treatment approach. Furthermore, residual cancer cells surviving therapy gradually divide, thereby initiating recurrence of the disease, often having worse responses to treatment and a poorer prognosis. Chemoresistance can develop through various mechanisms, such as gene mutation, DNA methylation, and histone modification (359). Numerous studies focus on identifying and understanding the role that miRNAs play in the development of chemotherapy resistance. Members of the miR-200 family appear to be critical of this phenomenon. Depending on cancer and the miR-200 family member, the underlying mechanism and the effect they have on modulating the resistance are different.

miR-200 family members reduce drug resistance in most cases (**Table 7**). miR-200a enhances drug resistance in breast and liver cancer, and it reduces drug resistance in glioma. miR-200b reduces drug resistance in 7 cancer types. miR-200c reduces drug resistance in 7 cancer types but it enhances chemoresistance in esophageal carcinoma. miR-141 increases drug resistance in breast cancer and glioma and it decreases drug resistance in neuroblastoma, colorectal and pancreatic cancer. miR-141 increases the resistance to cisplatin in non-small cell lung cancer and ovarian cancer. However, it reduces the resistance to other drugs in those cancer types. miR-429 reduces drug resistance in colorectal and ovarian cancers and increases drug resistance in endometrial cancer.

**Table 7:** Regulation of drug resistance by miR-200 family members.

miRNAs	Cancer	Target	Drug resistance	Ref.
miR-200a	Breast cancer	TP53INP1	↑	(360)
	Glioma	n/d	↓	(81)
	Liver cancer	DUSP6	↑	(361)
miR-200b	Bladder cancer	n/d	↓	(134)
	Cholangiocarcinoma	SUZ12 and ROCK2	↓	(51)
	Colorectal cancer	TUBB3	↓	(247)
	Non-small cell lung cancer	ATG12, E2F3, p70S6K1, SUZ12	↓	(254, 256, 362–365)
	Prostate cancer	n/d	↓	(262)
	Small cell lung cancer	ZEB2	↓	(366)
	Ovarian cancer	DNMT3A/DNMT3B	↓	(367)
miR-200c	Breast cancer		↓	(368)
	Gastric carcinoma	ZEB2	↓	(369)
	Melanoma	n/d	↓	(277)
	Osteosarcoma	AKT2	↓	(105)
	Ovarian cancer	DNMT3A/DNMT3B	↓	(367)
	Pancreatic cancer	n/d	↓	(330)
	Cholangiocarcinoma	SUZ12 and ROCK2	↓	(51)
	Esophageal carcinoma	n/d	↑	(370)
miR-141	Colorectal cancer	EGFR	↓	(291)
	Breast cancer	EIF4E	↑	(371)
	Glioma	TP53	↑	(372)
	Neuroblastoma	FUS	↓	(293)
	Non-small cell lung cancer	PDCD4	↑/↓	(365, 373)
	Ovarian cancer	KEAP1	↑/↓	(374, 375)
	Pancreatic cancer	MAP4K4	↓	(298)
miR-429	Colorectal cancer	DUSP4	↓	(376)
	Endometrial cancer	n/d	↑	(60)
	Ovarian cancer	ZEB1	↓	(377)

↓ - suppression by miRNA; ↑ - promotion by miRNA; n/d – no data.

MiR-200a demonstrates various effects on the modulation of drug resistance among different types of cancer. In breast cancer cells, Mir-200a, *via* antagonizing TP53INP1 and YAP1, contributes to increased resistance to chemotherapeutics (360). Moreover, inhibition of miR-200a enhances gemcitabine chemosensitivity in cancer cells (360). In human hepatocellular carcinoma, miR-200a-3p targets dual-specificity phosphatase 6 (DUSP6) to augment cancer cell resistance to 5-fluorouracil, doxorubicin, and cisplatin (361). In contrast, miR-200a has opposite effects on drug resistance in glioma. Moreover, downregulation of miR-200a is associated with decreased chemotherapeutic treatment efficacy (81).

MiR-200b appears to reduce the chemoresistance of all cancers. In cholangiocarcinoma, it was demonstrated that miR-200b, as well as miR-200c, reduces resistance to chemotherapeutics by directly targeting SUZ12 and ROCK2 (51). The same effect, but in a different mechanism, was reported with miR-200b-3p in colorectal cancer, where, *via* targeting TUBB3, it reduced resistance to oxaliplatin and promoted apoptosis and growth inhibition in resistant cancer cells (247). In bladder cancer cells, methylation of miR-200b was associated with resistance of this cancer to cisplatin (134). Moreover, it was suggested that epigenetic silencing of miR-200b might be a marker of cisplatin resistance in this tumor. In addition, miR-200b seems to play an essential role in the response of non-small lung cancer to treatment. It has been evidenced that induction of miR-200b, but also miR-141, increased sensitivity to nintedanib in nintedanib-resistant cells (365). Moreover, miR-200b increases the chemosensitivity of the docetaxel-resistant lung cancer cells by directly targeting autophagy-associated gene 12 (ATG12) (364). A further study revealed that expression of miR-200b, by direct targeting SUZ12 and through histone deacetylase 1/Sp1/miR-200b signaling pathway might lead to the formation of chemoresistant phenotype in docetaxel-resistant cancer cells. Moreover, histone deacetylase-mediated silencing of miR-200b increased chemoresistance in lung adenocarcinoma cells (254, 363). Furthermore, miR-200b in lung cancer cells inhibits chemoresistance and increased sensitivity to cisplatin *via* targeting p70S6K1 (256). This miRNA was also found to reverse the chemoresistance of docetaxel-resistant lung adenocarcinoma cells *via* targeting E2F3 (362). Moreover, also in small cell lung cancer, miR-200b reduces drug resistance namely by modulating ZEB2, which in small cell lung cancer leads to multidrug resistance of the tumor (366). By regulating B-cell-specific Moloney murine leukemia virus insertion site 1 (Bmi-1), miR-200b has been shown to enhance the chemosensitivity of prostate cancer cells to docetaxel (262). Besides, in ovarian cancer, miR-200b and miR-200c have been reported to be able to reverse cisplatin resistance by directly targeting DNMT3A/DNMT3B (367).

In most cases, miR-200c decreases drug resistance. In breast cancer, miR-200c increases sensitivity to chemotherapy as well as sensitizes HER2+ cancer cells to trastuzumab (368). Moreover, miR-200c, by directly targeting and thus downregulating ZEB2, increases the sensitivity of gastric cancer tissues to cisplatin (369). In melanoma cells, overexpression of miR-200c significantly reduces resistance to chemotherapy *via* downregulation of Bmi-1 (277). This miRNA was also revealed to enhance osteosarcoma chemosensitivity to cisplatin by targeting AKT2 (105). In pancreatic cells, miR-200c sensitizes cancer cells to chemotherapy (330). In contrast, overexpression of miR-200c in esophageal cancer induces chemoresistance to cisplatin by activation of the Akt signaling pathway (370).

Similarly, miR-141 has been reported to have significant effects on the modulation of drug resistance. In neuroblastoma cells, miR-141 increases cancer cell sensitivity to cisplatin (293). Also in pancreatic cancer, miR-141, by directly targeting MAP4K4, increases the chemosensitivity of the cancer cells (298). However, in breast cancer upregulation of miR-141 has been found to exacerbate docetaxel resistance of cancer cells (371). In non-small cell lung carcinoma, the function of miR-141 is more complex as, on the one hand, it increases sensitivity to nintedanib in nintedanib-resistant cancer cells (365), but on the other hand, *via* upregulation of PDCD4, it reverses cisplatin resistance (373). Moreover, in ovarian cancer, the action of miR-141 is also bidirectional. Transfection with inhibitors of miR141, as well as of inhibitors of miR-200c, in ovarian cancer cell lines induced cell resistance to paclitaxel and carboplatin (375); however, it has been described that expression of miR-141, *via* regulating KEAP1, can increase resistance to cisplatin chemotherapy in ovarian cancer cells (374). In glioma cells, miR-141 renders resistance to temozolomide therapy by targeting TP53 (372).

The last member of the miR-200 family is miR-429, whose effect on the modulation of drug resistance has been observed in colorectal, endometrial, and ovarian cancers. In colorectal cancer, overexpression of miR-429 has been found to target DUSP4, block the JNK pathway, and thereby increase cancer cell sensitivity to nintedanib (376). Also in ovarian cancer, overexpression of miR-429 appears to sensitize cancer cells to cisplatin by targeting ZEB1 (377). However, in endometrial cancer, the effect of miR-429 on drug resistance of this tumor is the opposite, as transfection with anti-miR-429 increased the cytotoxic effect of cisplatin in cancer cells, thus improving treatment efficacy (60).

## Regulation of immune response by miR-200 family

Tumor cells evade immune response *via* multiple mechanisms. Programmed Cell Death Protein 1 (PD-1) and its ligand, PD-L1, are key immune checkpoint molecules suppressing anti-tumor immune response (378). Several miRNAs were identified to regulate the PD-1/PD-L1 axis, including miR-200 (379). By targeting PD-L1, miR-200 enhances CD8^+^ cytotoxic T-cells activity in the tumor microenvironment and regulates the metastatic potential of tumor cells (379). Moreover, PD-L1 is targeted by miR-429 in gastric cancer (380).

MiR-200 family regulates also myeloid cells in the tumor microenvironment. MiR-200c by targeting PTEN and FOG2 induces the expansion and enhances the immunoregulatory properties of myeloid-derived suppressor cells (MDSCs) (381). It induces, among others, the expression of arginase 1, a key immunoregulatory enzyme of MDSCs (382), potentiating suppressive effects on T-cells (381). Moreover, miR-200c suppresses the expression of multiple cytokines by tumor-associated macrophages (383). Restoration of miR-200c upregulates cytokines and promotes M1 polarization of macrophages (383). Notably, cytokines suppressed by miR-200c predict favorable survival of TNBC patients (383). Similarly, miR-200a promotes phagocytosis of tumor cells by macrophages by targeting CD47, a “do not eat me” signal protein overexpressed in tumor cells (86). Nonetheless, more research is required to dissect the role of the miR-200 family in the antitumor immune response as well as patients’ response to immunotherapy.

## Genome instability and mutations

Various miRNAs regulate the expression of DNA damage proteins leading to genomic instability (384). However, little is known regarding the role of the miR-200 family in the regulation of genomic instability and mutations. It was demonstrated that miR-200a regulates DNA repair in aging keratinocytes (385), nonetheless, its role in genome instability in cancer requires further studies.

## Novel hallmarks of cancer

Recently, five emerging hallmarks of cancer have been suggested, including dysregulation of cellular energetic, unlocking phenotypic plasticity, dysregulation of the microbiome, nonmutational epigenetic reprogramming, and senescence (214). Despite little being known about the role of the miR-200 family in novel hallmarks of cancer, some studies suggested that they may be important regulators of these features.

MiRNAs, including the miR-200 family, are involved in the reprogramming of cancer cell metabolism. For instance, miR-200b suppresses lactate dehydrogenase A which suppresses glycolysis leading to the inhibited proliferation and invasion of glioma cells (386). Moreover, miR-200b/miR-200c regulates EMT differentiation and proliferation by modulation of metabolic properties of colorectal cancer cells (387). Notably, the miR-200 family also regulates the cellular senescence of cancer cells. It was demonstrated that suppression or loss of the miR-200 family in cancer cells induces morphological changes, cell cycle arrest, and induces cell senescence (388).

Recently, more and more studies have stated that the multifactorial impact of polymorphic microbiomes on cancer regulation is linked to their bidirectional interference with miRNAs in which those two factors interact resulting in apoptosis or proliferation of tumor cells (389, 390). A prominent example is an ovarian cancer where tissue-specific bacteria, *L. lactis*, seem to be in control of miR-200b and TLR-4 downregulation, which is connected to the progression of ovarian cancer (389). Another example of miRNA-microbiome interaction is exosomal miR-200c which was identified as a tumor suppressor of colorectal cancer cells, but only after it came in contact with lipopolysaccharide (LPS) that is a component of the bacterial outer membrane (391). Nonetheless, more research is required to determine the role of the miR-200 family in the regulation of these hallmarks of cancer.

## Conclusions

Members of the miR-200 family are crucial regulators of hallmarks of cancer. Several studies described alterations in their expression in human tumors and determined their utility as biomarkers in cancer. Our literature review summarized known functions and biological targets of the miR-200 family in different types of cancer. Furthermore, it identified gaps and limitations in current knowledge indicating the directions of further research. More studies are necessary to determine the role of members of the miR-200 family in the regulation of recently added hallmarks of cancer. Additionally, preclinical studies are required to determine the therapeutic potential of the miR-200 family.

## Author contributions

KK and TG are co-first authors. All authors listed have made a substantial, direct, and intellectual contribution to the work, and approved it for publication.

## Funding

This research was funded by the Medical University of Warsaw, grant number 1MN/1/M/MBM/N/21. The APC was funded by the Medical University of Warsaw. TG is supported by the PRELUDIUM grant funded by the National Center of Science (UMO-2021/41/N/NZ6/02774) and by the Foundation for Polish Science (FNP).

## Acknowledgments

Figures were created with BioRender.com.

## Conflict of interest

The authors declare that the research was conducted in the absence of any commercial or financial relationships that could be construed as a potential conflict of interest.

## Publisher’s note

All claims expressed in this article are solely those of the authors and do not necessarily represent those of their affiliated organizations, or those of the publisher, the editors and the reviewers. Any product that may be evaluated in this article, or claim that may be made by its manufacturer, is not guaranteed or endorsed by the publisher.
